# Multidimensional Characterization and Differentiation of Neurons in the Anteroventral Cochlear Nucleus

**DOI:** 10.1371/journal.pone.0029965

**Published:** 2012-01-09

**Authors:** Marei Typlt, Bernhard Englitz, Mandy Sonntag, Susanne Dehmel, Cornelia Kopp-Scheinpflug, Rudolf Ruebsamen

**Affiliations:** 1 Institute of Biology, University of Leipzig, Leipzig, Germany; 2 Complex Structures in Biology and Cognition, Max Planck Institute for Mathematics in the Sciences, Leipzig, Germany; Hotchkiss Brain Institute, University of Calgary, Canada

## Abstract

Multiple parallel auditory pathways ascend from the cochlear nucleus. It is generally accepted that the origin of these pathways are distinct groups of neurons differing in their anatomical and physiological properties. In extracellular *in vivo* recordings these neurons are typically classified on the basis of their peri-stimulus time histogram. In the present study we reconsider the question of classification of neurons in the anteroventral cochlear nucleus (AVCN) by taking a wider range of response properties into account. The study aims at a better understanding of the AVCN's functional organization and its significance as the source of different ascending auditory pathways. The analyses were based on 223 neurons recorded in the AVCN of the Mongolian gerbil. The range of analysed parameters encompassed spontaneous activity, frequency coding, sound level coding, as well as temporal coding. In order to categorize the unit sample without any presumptions as to the relevance of certain response parameters, hierarchical cluster analysis and additional principal component analysis were employed which both allow a classification on the basis of a multitude of parameters simultaneously. Even with the presently considered wider range of parameters, high number of neurons and more advanced analytical methods, no clear boundaries emerged which would separate the neurons based on their physiology. At the current resolution of the analysis, we therefore conclude that the AVCN units more likely constitute a multi-dimensional continuum with different physiological characteristics manifested at different poles. However, more complex stimuli could be useful to uncover physiological differences in future studies.

## Introduction

Neurons in the cochlear nucleus (CN), differing in their anatomical and physiological properties, give rise to different ascending parallel auditory pathways, each concerned with the processing of specific aspects of acoustic information [1]–[3] Insights into the functional organization of the CN are therefore essential for further comprehension of auditory brainstem processing.For the anteroventral division of the CN (AVCN) three principal morphological cell types have been described by Osen [4]: spherical bushy cells, globular bushy cells and stellate cells. Further subdivisions were postulated by Brawer et al. [5] and Lorente de Nó [6].

In parallel, electrophysiologists have commonly classified AVCN units *in vivo* based on their temporal responses properties to acoustic stimuli as seen in peri-stimulus time histograms (PSTH) [7]–[9] According to these studies, the major response categories in AVCN were termed *primary-like*, *chopper* and *onset* -units [7], where each category were subsequently further subdivided [8], [9] Still, it was shown that units exhibiting a specific PSTH also share other physiological characteristics such as spontaneous rate, inter-spike interval histograms, spectral response bandwidth, etc. [7]–[9].

Intracellular labelling of CN cells combined with *in vivo* recordings provided evidence for a relation between PSTH types and morphologically defined types of neurons [10]–[19]. The relationships that emanated from these studies are that *primary-like* patterns are attributed to spherical bushy cells, *primary-like with notch* patterns to globular bushy cells, and *chopper* patterns to stellate cells. However, even studies in favour of the respective physio-morphological correlation repeatedly reported cases that do not support the idea of equating both classifications. For example, Rhode [19] has recently shown a variation of different PSTHs for globular bushy cells, indicating that a classification which is exclusively based on PSTH types might not be sufficient for a comprehensive characterization of AVCN neurons.

In an earlier attempt, Blackburn and Sachs [9] have already evaluated a large number of physiological properties of AVCN units to define classifying boundaries between PSTH types. In our study, we followed along these lines, but made great efforts to not base the analysis on any presumptions regarding different physiological properties of the units. To achieve this goal, we used a multivariate statistic, which allows the classification based on a uniform evaluation of a large number of response properties. This enabled a much wider spectrum of physiological response properties to be taken into account and at the same time to consider potential relationships between all these parameters.

The data were collected from Mongolian gerbils, a model system that has gained importance over the last years in auditory research due to their distinct low frequency hearing ability. The vast majority of the previous studies investigating cell types in the AVCN have been conducted in cats, while only little work was done in gerbils [17], [20], [21]. So, the present report provides the first comprehensive examination of the physiological properties of neurons in the AVCN in the Mongolian gerbil.

We find that while the neurons of the same PSTH type are generally closer to each other than across PSTH types, no clear boundaries emerge and neurons of different PSTH type can be quite similar with respect to their physiological properties. We therefore conclude that at the present resolution of the analysis and the presently observed properties, the AVCN neurons appear to form a physiological continuum.

## Materials and Methods

All experiments were performed at the Neurobiology Laboratories of the Faculty of Bioscience, Pharmacy and Psychology of the University of Leipzig (Germany). The experimental procedures were approved by the Saxonian District Government Leipzig (TVV50/06) and conducted according to European Communities Council Directive (86/609/EEC).

### Animals and animal care

Adult pigmented (agouti) Mongolian gerbils (*Meriones unguiculatus*), aged 2–4 months and weighing 40–70 g, were used in the experiments. The animals were obtained from the animal care facilities of the Institute of Biology II of the University Leipzig. During the surgical preparation and the recording experiments, the animals were anaesthetized with an initial dose of a mixture of ketamine-hydrochloride (18 mg/100 g body weight, Ketavet®: Upjohn) and xyalzine-hydrochloride (0.5 mg/100 g body weight, Rompun®: Bayer). A constant state of anaesthesia was kept by supplementary injections of one-third of the initial dose (on average once per hour). Body temperature was maintained with a heating pad (Harvard Apparatus) at 37–38°C.

### Surgical preparation

For supporting the animal in a stereotaxic recording device the skull was exposed along the dorsal midsagittal line, and a small metal bolt was glued to the bone overlaying the forebrain. Two 500 µm diameter holes were drilled in the skull 2000–2300 µm caudal to the lambda suture. Through the first drill hole, located over the midline, the recording electrode was inserted. The second drill hole, located 1500 µm lateral to the midline, was used to position the reference electrode in the superficial cerebellum.

### Acoustic stimulation

Auditory stimuli were generated on a standard PC using custom written software (Rec_thor: M. Weick, University of Leipzig; and Spike: B. Warren, University of Washington, Seattle). The signals were then transferred to a D/A converter (RP2.1 Enhanced Real Time Processor, 97.7 kHz sampling rate, Tucker-Davis Technology; or DD1, 50 kHz sampling rate, Tucker-Davis Technology). Near-field acoustic stimuli were delivered through custom made earphones (acoustic transducer: DT 770 Pro, Beyerdynamic) fitted with probe tubes (plastic, 70 mm length, 4 mm diameter) which were placed close to the opening of the ear canal. The speakers were calibrated as described previously [22]. The maximum intensity used in the recording experiments was 90 dB sound pressure level (SPL).

### Recording setup

All experiments were performed in a sound-attenuated and electrically isolated chamber (Type 400, Industrial Acoustics Company). The animals were placed on a vibration-isolated table (T 251.SL, Physik Instrumente) and fixed in a custom made stereotaxic device by means of a metal bolt. Recording electrodes were glass micropipettes (GC150F-10, Harvard Apparatus) filled with 3 M KCl. Targeting the AVCN, the recording electrodes were advanced through the cerebellum and the brainstem with a piezo manipulator (PM101, Newport).

The extracellular recordings were amplified and filtered (Modell 1600, Neuroprobe Amplifier, A-M SYSTEMS; PC1, Tucker-Davis Technology [0.4 kHz high-pass filter and 7 kHz low-pass filter]; HumBug, Quest Scientific [50 Hz notch filter]). For isolation of single units, action potentials thresholds were set at voltages of at least two times the noise envelope (corresponding to approx. 4 times the S.D. of the noise), and the crossing of this level was taken as the time of occurrence of action potentials. The crossing of the threshold level triggered TTL-signals (SD1, Tucker-Davis Technology) which were digitized (RP2.1 Enhanced Real Time Processor, Tucker-Davis Technology; or ET1, Tucker-Davis Technology) and stored on a standard PC for offline analysis. Simultaneously, the voltage signals were digitized (DD1, 50 kHz sampling rate, Tucker-Davis Technology) and stored on the PC.

### Multiunit mapping

At the beginning of each experiment, the stereotaxic coordinates of the exact position and extent of the AVCN were determined by on-line analysis of acoustically evoked multiunit activity recorded with low-impedance electrodes (<5 MΩ). In several electrode penetrations we tested for neuronal responses evoked by ipsilateral acoustic stimulation. The characteristic frequency of multiunits was systematically documented every 200 µm along the dorsoventral dimension and every 200 µm in the rostrocaudal and also in the mediolateral dimension (2° change in angle of electrode penetration). Referring to the known tonotopic organization of the cochlear nucleus [23]–[27] it was possible to identify the exact penetration coordinates for the CN and its subregions in each experimental animal.

### Single unit recordings

The response properties of single units were examined with high-impedance micropipettes (10–30 MΩ). Single units were identified with regard to the bipolar shapes of their waveforms and signal constancy.

#### Frequency tuning curve and related response characteristics

A unit's response area was measured by presenting pure-tone pulses (100 ms duration, 5 ms rise-fall time, 100 ms inter-stimulus interval) within a predefined matrix of 16×15 frequency/intensity combinations. Stimuli were presented five times in a pseudo-random order. Spontaneous activity was acquired in silent runs interspersed with the stimulus runs. The number of spikes was measured during the 100 ms period of stimulus presentation. From these data characteristic frequency (CF, frequency with the lowest threshold), best frequency (BF, frequency with maximum discharge rate), and their difference (CF-BF) were obtained. Additionally, the following sound-evoked response characteristics were computed: threshold at CF (TRS), spontaneous (spontR) and maximum discharge rates (maxR), rate-level function at CF (RLF), dynamic range (DR), response bandwidth 10 dB above the unit's threshold (Q_10_-value, Kiang 1965), the slope of the frequency tuning curve at the respective high-frequency and low-frequency borders sites, and the occurrence of inhibitory sidebands. For the categorical properties RLF and inhibitory sidebands we translated each category to a number in order to include them in the cluster analysis. For RLF the assignments were strictly monotone to 1, monotone-plateau to 2, and non-monotone to 3. For inhibitory sidebands we assigned units that show no sidebands to 0, units that show a sideband at the low-frequency side of the FTC to 0.8, units with a sideband at the high-frequency side to 0.9 and units with both, low- and high-frequency sidebands, to 1.

#### Peri-stimulus time histogram (PSTH)

PSTHs were acquired in response to pure tone pulses (100 ms duration, 5 ms rise-fall time, 100 ms inter-stimulus interval, 50 repetitions) at the unit's CF and with 80 dB SPL.

The classification of the PSTHs was based on the definitions by Pfeiffer [7], Rhode and Smith [8], Young et al. [28] and Blackburn and Sachs [9]. *Primary-like* (PL) PSTHs display phasic-tonic discharge patterns, similar to those obtained from auditory nerve fibres. *Primary-like with notch* (PL_N_) PSTHs are similar to the PL ones, but here the initial response peak is separated from the subsequent tonic activity by a short (<2 ms) pause. *Chopper* PSTHs exhibit several regularly spaced peaks with interpeak distances unrelated to the stimulation frequency. The *chopper* units were subdivided into two subcategories: *transient choppers* (C_T_) which display the regular discharge pattern only at the beginning of the response, while in *sustained choppers* (C_S_) prominent periodic discharge peaks were found throughout the entire duration of the response. To differentiate between C_T_ and C_S_ units we used the coefficient of variation (CV) as introduced by Young et al. (1988). Units with CVs smaller than 0.35 were classified as C_S_, units with larger CVs as C_T_. For the cluster analysis the following parameters were quantified from the PSTH: peak-over-total value as the number of APs occurring during the stimulus vs. the number of APs in the onset peak (all 0.5 ms bins around the maximum peak with at least 2/3 height of the maximum peak), average inter-spike interval (ISI) with standard deviation (S.D.), the average first spike latency (FSL), and the jitter of the first spike.

For the ISIs and their standard deviation only the first 20 ms of the stimulus are consider for better comparability with previous studies. We are aware that the ISI most likely correlate with the maximum discharge rate. However, the maximum discharge rate corresponds to the best frequency and the ISI to the characteristic frequency; thus both parameters could potentially carry different information and therefore were both included in the analyses.

For the FSL the first spike of each trail is included in the analysis. Note, that the dependency of first spike latency on CF is implicitly taken into account by the multidimensional analysis, where covaritions between difference parameters would produce elongated clusters, which could be identified by the hierarchical cluster analysis we employed.

Due to the often limited single-unit recording time we had to restrict the recordings to the minimum. We therefore choose to use only one stimulus level for evaluating PSTHs. We decided to use 80 dB SPL for several reasons: (i) A threshold independent stimulus level avoids presumptions about connections between single parameters. Later on a correspondence to the threshold is always possible and was considered in the cluster analyses, since there all parameters are considered in parallel. (ii) At 80 dB SPL most of the units responded close to their maximal discharge rate and thus in a comparably activated state. We are aware that there are few units for which 80 dB SPL lies still in their dynamic range (especially those with high thresholds) and some units which already decrease their response at this level (units with non-monotonic RLF). (iii) The shape of the PSTH is more likely to be fully developed at high levels. For example, Blackburn and Sachs [9] showed for some cases that the notch becomes only visible for higher stimulation levels and would hence influence the differentiation between PL and PLN.

#### Sinusoidal amplitude modulation (SAM)

Temporal encoding of rapid amplitude fluctuations in the stimuli was quantified from the responses to sinusoidally amplitude-modulated tone bursts at CF, 20 dB above threshold. Modulation depth of the SAM signals was 100% with modulation rates from 20 Hz to 1000 Hz (stimulus duration: 100 ms, inter-stimulus interval: 100 ms, 50 repetitions). To exclude a contamination of the units' SAM coding by the onset response, only the steady-state response (10–100 ms) was included in the analysis. The data were quantified by calculating the vector strengths (VS) and entrainment (entr) of neuronal discharges [29]–[32].

#### Waveform analysis

The waveforms of neuronal discharges were collected by triggering the voltage trace at a visually determined, conservative level. The waveforms of the triggered potentials were collected in intervals from 2 ms preceeding to 2.5 ms following the trigger. The average waveforms of the units were then used to quantify the signal-to-noise ratio (SNR, the positive peak of the AP divided by the standard deviation of the noise) of the respective single unit recording and the time between the maximum and minimum amplitude of the bipolar signals as an indicator of the duration of the unit's AP (t(AP)). The averaged waveforms were also inspected for distinct P, A, and B components as described for spherical bushy cells [33]–[35].

### Verification of recording sites

The recording sites were verified histologically in 6 animals using horseradish peroxidase (HRP, Sigma) and in 10 animals using biotinyled dextranamin (BDA, Moleculare Probes). HRP was injected iontophoretically (+1.5 µA, 4–5 min) at the recording site; BDA was applied by pressure injections. The animals were allowed to survive for 24 h or one week, respectively. Then, they were given a lethal dose of Na-pentobarbital (100 mg/100 g body weight i.p, Narcoren®, Merieux) and perfused via the left ventricle of the heart with 0.9% NaCl solution followed by fixative (2.5% paraformaldehyde in 0.1 M phosphate buffer, pH 7.4). The brains were removed from the skull and post-fixed in paraformaldehyde, and thereafter embedded in agarose. Serial transverse sections of the brains (50 µm) were cut using a vibratome. BDA brain sections were reacted using an avidin-HRP complex (Molecular Probes). Accordingly, the BDA sections as well as the HRP sections were treated with 3,3′-diaminobenzidine to visualize the HRP (Adams 1981). The HRP sections were counterstained with cresyl violet (Nissl stain). Electrode tracks and recording sites were reconstructed by examining the sections with the light microscope.

### Statistics

For statistical analysis SigmaPlot 8.0, SigmaStat 3.0 (both SPSS inc.) and MATLAB 7.3 (Mathworks Inc.) were used. If data were normally distributed (Kolmogorov-Smirnov-test) the results are displayed as means±S.D., otherwise as medians and the respective 25% and 75% quartiles. The statistical significance (p<0.05) of differences between groups was assessed using one way ANOVA followed by Holm-Sidak *post-hoc* test if the data was normally distributed. For data with other distributions a one way rank-based ANOVA followed by a *post-hoc* test according to Dunn's method was used.

#### Multivariate statistics

To test whether the sample can be divided into groups based on electrophysiological response properties, hierarchical cluster analysis was employed. This method allows a classification on the basis of a wide spectrum of parameters simultaneously. The number of expected groups does not need to be predefined, thus the result is not affected by prior assumptions. Before performing the cluster analysis all parameters were standardized to remove the effect of scaling differences between parameters. Dissimilarities between units were expressed as distances (linkage distance) in a space of as many dimensions as the parameters taken into account. In the present study *Euclidean distance* between objects was used. It is the most commonly used distance and simply figures the geometric distance in the multidimensional space. Classifications based of other distance metrics yielded qualitatively similar results. For joining smaller clusters into larger ones *Ward's method* was used, which attempts to minimize the variance within the groups [36].

**Figure 1:**
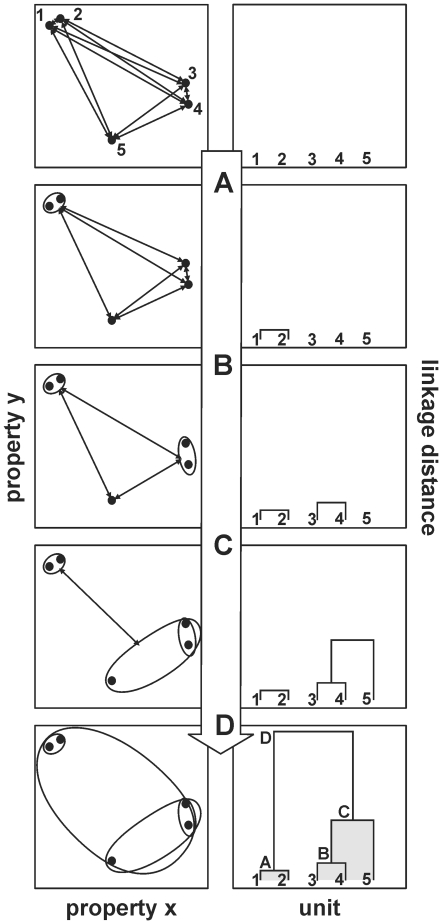
Schematic of aggregation procedure in hierarchical cluster analysis. **Left column**: The position of single units in the n-dimensional space. In each step (A–D) units or groups of units with the smallest distance to each other are grouped together. **Right column**: The resulting developing dendrogram of the cluster analysis. The linkage distance indicates the similarity of the units. The higher the linkage distance the more differ the units in their properties.

Figure 1 shows the aggregation procedure schematically. On the left side the units are presented as points in the multidimensional space (here only two dimensions). On the right side the developing dendrogram is shown for every aggregation step. It starts with the individual cells at the bottom. First, the two closest units in the multidimensional space were grouped together. Then, at each step, the number of groups is reduced by merging the two groups or units whose combination gives the least increase in the within-group sum of squared deviation. The linkage distance is a parameter for the heterogeneity of the joined groups. When linkage distance increases, branch points represent clusters of increasing size and dissimilarity. The final number of groups is determined at the stage where the maximal decrease in linkage distance is observed.

To illustrate the position of the single units in relation to each other, a principal component analysis was performed. Principal component analysis projects the units from the multidimensional space to a space of lower dimensionality while attempting to preserve the distance relations between them. Therefore the multidimensional space was rescaled in a way that the first new coordinate (principal component 1, PC1) goes through the greatest variance by any projection of the data, the second coordinate (principal component 2, PC2) through the second greatest variance, etc. Besides suggesting a grouping of the sample, this method also indicates which set of parameters best explains the dissimilarity between groups.

Note, that both hierarchical cluster analysis and principal component analysis can aid in uncovering structure in data sets and suggest a scheme for classification. Yet, they do not produce a measure of statistical significance for differences between suggested groups. To verify our results we employed (i) different subpopulations of our unit sample and (ii) different sets of parameters and compared the respective cluster analyses. Changes in the sample size and in the number of parameters should be tolerated by the cluster analysis as long as the sample provides enough information of the whole population and the chosen parameters provide enough potential to distinguish the population. Otherwise the analysis would result in small, only minimal separated groups. To avoid this, our analysis is based on a broad spectrum of parameters that potentially could separate the neuron population. Thereby we tried to not exclude parameters that, at first, seem not to have a major impact on the classification and also not to include parameters twice, in form of different expressions (e.g. the CV-value additionally to ISI mean and ISI standard deviation). We also aimed to include the parameters in their most raw form to avoid distortions.

## Results

The analyses were based on data acquired from 233 units recorded in the AVCN of Mongolian gerbils. The minimum requirement for a data set to be included in the analysis was a complete acquisition of a unit's response area based on recordings during tone burst presentations (5 repetitions of 16×15 frequency-intensity combinations) and 16 sec (5×16×200 ms) recordings of spontaneous discharge activity. For the majority of the units (n = 181, 78%) additional recordings with 50 stimulus repetitions at the respective characteristic frequency (CF) could be achieved, which enabled a refined analysis of the unit's temporal response characteristics. Furthermore, for 60 units (26%) the discharge activity to sinusoidal amplitude modulated signals (SAM) was registered for modulation frequencies from 20 to 1000 Hz. The waveform of the extracellular recorded single unit field potential was digitized in 154 units (66%). This data set formed the basis for the analysis of the units' frequency and sound level coding as well as their temporal coding (detailed response properties for all units are summarized in table 1).

**Table 1:** Physiological response properties of different PSTH groups in AVCN.

response properties	PSTH-group^a^				Statistical analyses	
	PL	PL_N_	C_T_	C_S_	ANOVA	*Post-hoc* test^b^
**Frequency tuning curve**	**n = 57**	**n = 48**	**n = 65**	**n = 37**		
CF [kHz]	2.3 (1.6; 7.8)	18.1 (10.1; 23.	15.2 (2.6; 29.0	14.6 (7.3; 19.6)	P<0.001	(PL) (PLN CT CS)
TRS [dB]	32±22	34±15	28±18	23±19	P<0.05	(PL PLN CT) (PL CT CS)
CF-BF [octaves]	0 (0;−0.66)	0.21 (0; 1.08)	0.09 (0; −0.90)	0 (0;−0.29)	P = 0.197	(PL PLN CT CS)
BF-CF [Hz]	8 (0; 25)	8 (0; 29)	14 (0; 47)	4 (0; 15)	P = 0.090	(PL PLN CT CS)
spontR [Hz]	32 (3; 59)	1 (0;27)	0 (0; 17)	7 (0; 26)	P<0.001	(PL) (PLN CT CS)
maxR [Hz]	162 (126; 216)	183 (140; 220)	298 (255; 365)	410 (284; 486)	P<0.001	(PL PLN) (CT CS)
Q_10_	2.11 (1.47; 2.80)	2.28 (1.57; 3.47)	1.89 (1.25; 3.29)	2.20 (1.38; 3.23)	P = 0.772	(PL PLN CT CS)
DR [dB]	30 (20; 44)	30 (25; 45)	40 (30; 55)	45 (35; 51)	P<0.001	(PL PLN) (CT CS)
difference of slopes [octaves/10 dB]	0.16 (0.06; 0.25)	0.12 (0.03; .23)	0.07 (0.08; 0.36)	0.02 (0.01; 0.18)	P<0.05	(PL PLN CT) (PLN CT CS)
**PSTH**	**n = 34**	**n = 42**	**n = 58**	**n = 36**		
peak-over-total [%]	9.8±4.9	6.3±4.3	8.4±3.4	13.8±4.1	P<0.001	(PL CT) (PLN) (CS)
ISI mean [ms]	3.4 (2.8; 4.8)	3.8 (3.1; 4.6)	2.6 (2.2; 3.1)	2.0 (1.6; 2.8)	P<0.001	(PL PLN) (CT CS)
ISI S.D. [ms]	2.18 (1.7; 2.4)	2.40 (1.6; 2.9)	1.34 (1.0; 2.0)	0.58 (0.4; 0.9)	P<0.001	(PL PLN) (CT) (CS)
FSL mean [ms]	4.50 (4.0; 6.6)	3.76 (3.3; 4.7)	3.88 (3.4; 4.3)	3.88 (3.4; 4.3)	P<0.01	(PL) (PLN CT CS)
FSL jitter [ms]	1.15 (0.6; 2.1)	0.44 (0.2; 0.9)	0.25 (0.2; 0.8)	0.39 (0.2; 0.7)	P<0.001	(PL) (PLN CT CS)
**SAM**	**n = 18**	**n = 13**	**n = 16**	**n = 10**		
VS max	0.52±0.16	0.63±0.16	0.69±0.13	0.72±0.14	P<0.01	(PL PLN) (PLN CT CS)
fmod VSmax [Hz]	50 (20;100)	200 (88; 625)	200 (150; 200)	200 (200; 300)	P<0.01	(PL) (PLN CT CS)
fmod VS>0,3 [Hz]	400 (200; 600)	1000 (575; 1000)	500 (400; 750)	650 (400;900)	P<0.05	(PL CT CS) (PLN CT CS)
entr max	0.59±0.15	0.72±0.12	0.79±0.13	0.93±0.09	P<0.001	(PL) (PLN CT) (CS)
fmod entr max [Hz]	100 (50; 200)	200 (100; 200)	200 (200; 300)	300 (300; 400)	P<0.001	(PL PLN) (CS CT)
**Waveform**	**n = 23**	**n = 37**	**n = 51**	**n = 32**		
SNR	7.4 (6.3; 9.1)	6.3 (5.5; 7.6)	8.4 (6.9; 11.0)	12.0 (7.9; 14.5)	P<0.001	(PL PLN) (PL CT) (CT CS)
t(AP) [ms]	0.26 (0.23; 0.32)	0.32 (0.27; 0.36)	0.28 (0.23; 0.32)	0.28 (0.23; 0.35)	P<0.05	(PL PLN CT CS)

abbreviations explained in methods.

categorical values are described in text.

a Means ± S.D./Median (25%, 75%).

b PSTH groups that do not significantly differ are within one parentheses.

In analysing the data we used two strategies. First, we grouped the data based on temporal response patterns into the ‘classical’ AVCN PSTH groups and tested for co-variations with single other response features. Second, we performed a cluster analysis using all evaluated parameters.

### Sample characteristics

This first part of the results gives an overview of the properties of the recorded AVCN units based on classical classification criteria which relate to current models of distinct pathways of auditory signal processing. These models proceed on the assumption of neurons in the AVCN with distinct morphological and physiological properties: spherical bushy cells, globular bushy cells and stellate cells. However, the variability of the physiological properties within the neuron population highlights the necessity of the cluster analysis presented subsequently.

#### Temporal response patterns

**Figure 2:**
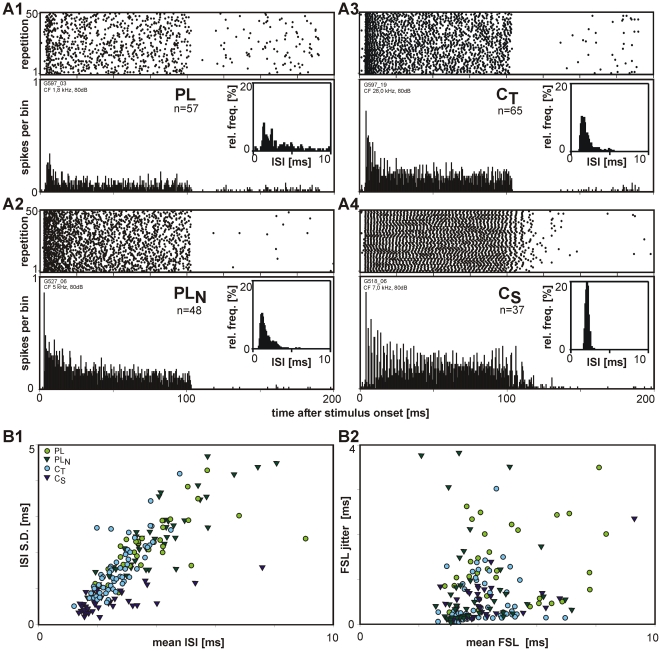
Classification of AVCN units based on PSTH types. **A1–A4**: Response patterns during tone burst stimulation (100 ms, 80 dB SPL at CF) of four units; dot raster to 50 stimulus presentations above, PSTH below (bin width 0.5 ms). **A1**: *primary-like* (PL), **A2**: *primary-like with notch* (PL_N_), **A3**: *transient chopper* (C_T_), **A4**: *sustained chopper* (C_S_). The insets show the inter-spike interval (ISI) distribution during the first 20 ms of the stimulus of the respective units (bin width 0.1 ms); **B1**: Relation between mean ISI and S.D. of ISI for the different unit types (symbols as indicated in the figure) **B2**: Relation between the first spike latency (FSL) and the jitter of the FSL. Note that both types of *chopper* units have lower ISIs and a lower variation in ISI than PL and PL_N_ units. Shortest latencies are observed in PL_N_ units.

In previous studies the temporal response characteristic was an important feature for classifying CN units [7]–[9]. Referring to this classification, which is based on the PSTH of responses to CF tone bursts at 80 dB SPL, our AVCN unit sample could be subdivided into 6 classes, 4 of which are shown in figure 2A. One quarter of the units (n = 57, 25%) showed a *primary-like* (PL) response pattern. *Primary-like with notch* (PL_N_) responses were obtained in 20% (n = 48), *transient chopper* (C_T_) responses in 28% (n = 65), and *sustained chopper* (C_S_) in 16% (n = 37) of the units. Fourteen units (6%) displayed an *onset-inhibitory* response (O_inh_), characterized by a sharp onset peak followed by strongly reduced discharge activity which was even below the spontaneous rate (data not shown). Only a fraction (5%, n = 12) of the units phase-locked to pure tones at their CF up to 1 kHz. Such phase-locking made it difficult to assign these units to one of the before mentioned PSTH types; therefore they were assigned in a separate group (PHL). In the following analysis we focus only on the four main PSTH types, since the data sets of the O_inh_ and PHL units are too small for statistical analyses.

In all PSTH types the peak-over-total ratio varied widely from 1% to 20%, but C_S_ units had the highest peak-over-total ratios among the units of the four main PSTH types (13±4%). In PL units the peak-over-total ratio ranged from <1% to 19% (10±5%), in PL_N_ from 1% to 22% (6±4%), and in C_T_ from 1% to 19% (8±3%).

The mean inter-spike intervals (figure 2B1) of both types of *chopper* units (C_S_: 2.0 ms [1.6 ms; 2.8 ms]; CT: 2.6 ms [2.2; 3.1]) were significantly smaller than those of PL (3.4 ms [2.8; 4.2]) and PL_N_ units (3.9 ms [3.1; 4.6]). Compared to the other units, both types of *chopper* units also showed a tendency for a smaller variance in inter-spike intervals (C_S_: 0.6 ms [0.4; 0.9], C_T_: 1.3 ms [1.0; 2.0], PL: 2.2 ms [1.7; 2.4], PL_N_: 2.4 ms [1.6; 2.9]). Despite these differences the respective values overlap strongly in the different PSTH types.

The first spike latencies (figure 2B2) were longest in PL (4.5 ms [4.0; 6.6]) units. Also the jitter of the first spikes was larger in PL units (1.15 ms [0.6; 2.1]) than in units of other PSTH types. The values of PL_N_, C_T_, and C_S_ units do not differ significantly. For none of the PSTH a dependency of the first spike latency from the characteristic frequency could be found, also there was a slight trend to shorter latencies at higher frequencies.

In summary these results show that the consideration of the PSTH type, the statistics of the inter-spike intervals and of latencies of the responses to acoustic stimuli does not lead to an unambiguous separation of the unit sample.

#### Waveforms of action potentials

**Figure 3:**
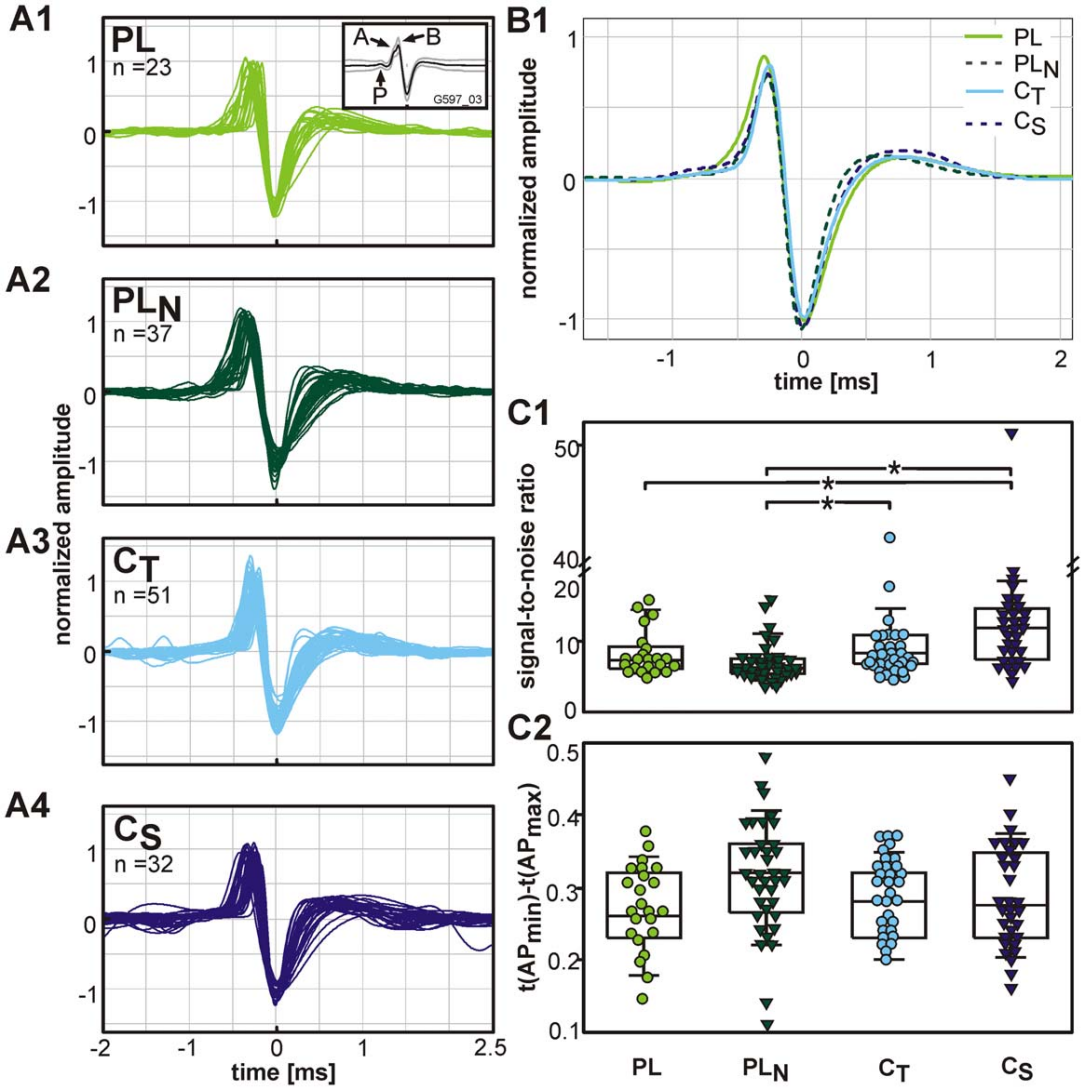
Waveform analysis. **A1–A4**: Averaged and normalized waveforms of single units sorted according to their PSTH types (numbers of units indicated in the graphs). The inset in A1 shows the mean waveform of the action potentials (black line, grey lines: S.D.) of a PL unit displaying the presynaptic component P and the two postsynaptic components ‘A’ and ‘B’. **B1**: Normalized average waveforms of all units of the respective PSTH types. **C1**: Signal-to-noise ratio and **C2**: duration from the maximum to the minimum of the waveforms of the respective PSTH types. Each symbol represents the value of a single AVCN unit. Significant differences (p≤0.05) between PSTH types are indicated with asterisks.

The spike discharges acquired with electrolyte-filled glass micropipettes typically displayed bipolar waveforms (figure 3). Complex waveforms composed of a presynaptic component (P) and two postsynaptic components (A/EPSP+B/postsynaptic AP, inset figure 3A1) [33], [35] were observed in 17% (4/23) of the PL units and in one PL_N_ unit. These units exclusively responded to low frequencies (CF<2 kHz). Only 2/7 units in this CF range lacked the presynaptic component. No such presynaptic components were seen in units with higher CFs. Still, in 32% (11/37) of the PL_N_ units postsynaptic EPSP (A-component) and AP (B-component) could be separated in the averaged AP waveforms; the same holds for 8% (4/51) of the C_T_ and 12% (4/32) of the C_S_ units.

The signal-to-noise ratios of the recordings of the whole unit sample varied between 5.2 and 16 with systematic differences in different PSTH groups (figure 3C1). The highest signal-to-noise ratios were obtained in C_S_ units (12.0 [7.9;14.5]), which were significantly higher than in PL (7.4 [6.3; 9.1]) and PL_N_ units (6.3 [5.5; 7.6]). The C_T_ units (8.4 [6.9; 11.0]) also had significantly higher signal-to-noise ratios than PL_N_ units.

However, the duration of the APs did not significantly differ between the different PSTH groups (figure 3C2). This was inferred from measuring the time between the maximum and minimum of the bipolar APs signals which range from 0.11 to 0.48 ms. The data show only a slight tendency for PL_N_ units to have longer APs.

#### Tuning characteristics and spontaneous activity

**Figure 4:**
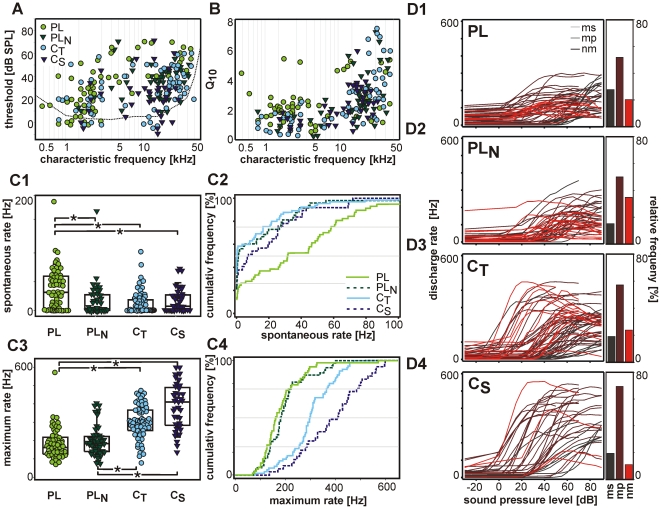
Tuning characteristics and spontaneous activity. **A**: Distribution of characteristic frequencies and threshold values for units of different PSTH type (dotted line: behavioural audiogram of the Mongolian gerbil, Ryan 1976). **B**: Frequency selectivity of the units quantified by Q_10_-values (symbols as in A). **C1 and C3**: Distribution and **C2 and C4**: cumulative frequency of the spontaneous (**C1 and C2**) and of the maximum (**C3 and C4**) discharge rates of the respective PSTH types. Note that PL units have the highest spontaneous but low maximum discharge rates. Highest discharge rates were observed in C_S_ units. **D1–D4**: Rate-level functions of the units of different PSTH types. The histograms on the right side of each graph show the fraction the different rate-level function types: steady monotonic (ms), monotonic with plateau (mp), non-monotonic (nm). Note that all types of rate-level functions have their share in the different PSTH groups.

The characteristic frequencies (CF) of the units covered the range from 0.3 to 45 kHz (figure 4A). In all PSTH groups the CFs spread widely, and the only significant difference was between the lower average CF in PL units (2.3 kHz [1.6; 7.8]) and the rest of the unit types (PL_N_: 18.1 kHz [10.1; 23.0]; C_T_: 15.2 kHz [2.6; 29.0], C_S_: 14.6 kHz [7.3; 19.6]). As a whole, the threshold values did not differ between the PSTH groups (means: 20–35 dB SPL). Only in the subgroup of units with CFs>10 kHz the thresholds of PL units were elevated (55 dB SPL [43.75; 66.25]) in comparison to the other PSTH groups.

Units of all PSTH groups displayed V-shaped frequency threshold curves with low-frequency tails. The slopes of the low-frequency flanks of the tuning curves were typically less steep than the slopes of the high-frequency flanks. The respective maximal values were 1.64 octaves and 1.15 octaves frequency increase per 10 dB. There was a tendency for C_S_ units to have more symmetric frequency tuning curves than units of other PSTH groups. In C_S_ units the average difference between the slopes of the high- and the low-frequency flank was only 0.02 octaves/10 dB. In contrast, it measured 0.16 octaves/10 dB in PL units.

The unit's frequency selectivity was quantified by Q_10_-values. In all PSTH groups Q_10_-values correlate strongly with the unit's CF (figure 4B). High-CF units show larger Q_10_-values, i.e. better frequency selectivity than low-CF units. When comparing units within specified CF-ranges, PL units showed the best frequency selectivity.

Inhibitory sidebands could only be observed in units with sufficiently high spontaneous discharge rates (>10 Hz). Among those, the incidence of inhibitory sidebands was highest in C_S_ (77%; 14/18) and C_T_ units (62%, 15/24), followed by PL (58%; 23/41) and PL_N_ units (42%; 8/19).

In most units (82%, 190/233), the CF was within the ±1 octave range of the best frequency (defined as the stimulus frequency eliciting the highest stimulus evoked discharge rate). In 42 of the remaining 44 units the best frequency was below CF by up to 5 octaves. No apparent differences were observed between the PSTH groups. However, the units' maximum discharge rates were significantly higher in *chopper* units (C_S_: 410 Hz [284; 486], C_T_: 298 Hz [255; 365]) than in PL (163 Hz [126; 216]) and PL_N_ units (183 Hz [140; 220]). Still, as for the CFs, there was a wide overlap of the respective values between different PSTH types (figure 4C3+4).

The spontaneous discharge rates of PL units (32 Hz [3]; [59]) were significantly higher than in any other PSTH group (figure 4C1+2). In PL_N_ (0 Hz [0; 27]), C_T_ (0 Hz [0; 17]), and C_S_ (7 Hz [0; 26]) units the distribution of spontaneous rates was mostly in the same range.

Three different types of rate-level functions (RLF) were distinguished (figure 4D1–4): monotonic RLF in which the discharge rates increase with increasing intensity level without saturation (strictly monotonic, ms, indicated as light grey in figure 4D); monotonic RLF which saturates at a certain level (monotonic-plateau, mp, medium grey), and non-monotonic RLF (nm, dark grey), in which the discharge rates reach a maximum in the mid-level range and then decrease towards higher intensities. In all PSTH groups, monotonic-plateau RLFs were the most frequent ones (50–69%), the monotonic and the non-monotonic RLFs had shares of 19–28% and 11–35%, respectively. The dynamic ranges differed between the PSTH groups: PL (30 dB [20]; [44]) and PL_N_ units (30 dB [25]; [45]) had significant smaller dynamic ranges than *chopper* units (C_T_: 40 dB [30]; [55], C_S_: 45 dB [35]; [51]). However, these differences did not circumscribe clear borders between the PSTH groups.

#### Response to sinusoidal amplitude modulations (SAM)

**Figure 5:**
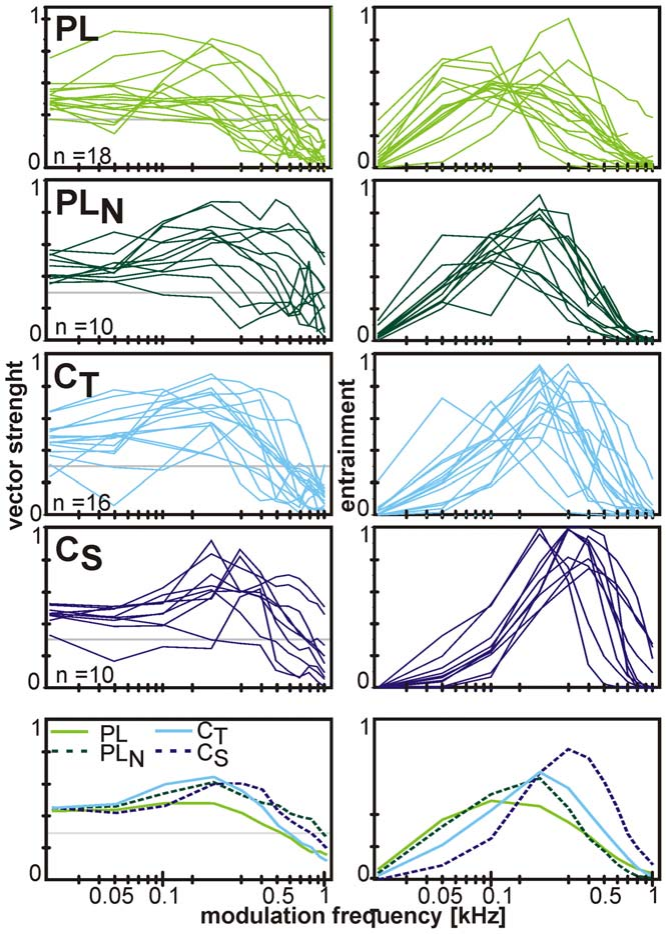
Responses to SAM. Synchronization indices (left column) and entrainment (right column) as a function of modulation frequency for the different PSTH types. The respective bottommost plots show the average transfer functions for each PSTH type. The horizontal line indicates the 0.3 cut-off criterion that was chosen to classify responses as being phase-locked. Note that C_S_ units show best and PL units worst ability to comodulate with fast fluctuations in stimulus amplitude.

To quantify the ability of AVCN units to respond to rapid fluctuations in signal amplitude, the vector strength and the entrainment to SAM stimuli varying in modulation frequencies (f_mod_) from 20 to 1000 Hz were calculated (figure 5).

A vector strength value of 0.3 was chosen as a cut-off criterion for classifying a response to SAM as being phase-locked. On average, PL units phase-locked to SAM stimuli up to 400 Hz (200; 600), while PL_N_ units phase-locked up to 1000 Hz (575; 1000). In PL units also the maximum vector strength values were lowest (0.52±0.16) and they were obtained at low f_mod_ (50 Hz [20; 100]). The highest vector strength values were found in C_S_ units (0.72±0.14) at higher f_mod_ (200 Hz [200; 300]). Also the entrainment, i.e. the proportion of SAM cycles in which at least one spike was recorded, reached the highest values and encompassed the highest modulation rates in C_S_ units (0.93±0.09 at 300 Hz [300; 400]). Again, the lowest respective values were found in PL units (0.59±0.15 at 100 Hz [50; 200]). Both SAM phase-locking and entrainment showed large overlaps between the PSTH groups.

In summary, a separate consideration of different parameters for frequency, sound pressure and temporal coding by AVCN units did not lead to an unambiguous separation of physiologically defined unit types. In fact, there is a large variability of values within each PSTH group and a strong overlap between the groups. Therefore we pursued an alternative approach and analysed the whole data set in a multidimensional parameter space.

### Cluster analysis

The following cluster analyses are based on the same sample of AVCN units and the same set of discharge properties described above. Cluster analysis can uncover structures in data sets and suggest a scheme for classifying the unit sample. It may result in the identification of distinct groups of units with a fixed set of distinguishing properties, or in identification of groups of units which differ in their properties, but which are not strictly separated from each other, or alternatively in the absence of distinct groups (continuum).

#### Classification of AVCN units considering all evaluated properties

To allow a complex physiological classification of AVCN units, all 22 evaluated properties were included in this analysis. For the cluster analysis an equalled sample, i.e. the same number of parameters for each unit is required. To sustain a sufficient sample size missing values, i.e. values that could not be acquired because of limitations of single unit recording time, were substituted by the mean of the respective property in the whole unit sample.

**Figure 6:**
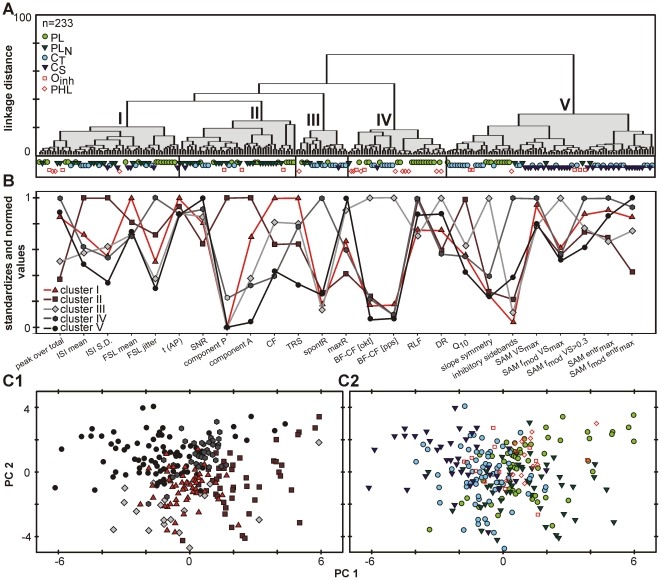
Cluster analyses considering all evaluated response properties. **A**: Dendrogram illustrating the result of hierarchical cluster analysis. The units (n = 233) are lined up at the bottom of the graph. The analysis suggests five clusters characterized by a specific distribution of parameter values. **B**: Mean of the respective parameter values for each property in the resulted clusters I–V. The values are standardized and normalized to the respective maxima (for original data and statistical analyses see table 2). Note that for almost all individual properties significant differences exist between the clusters, and some properties also correlated across clusters. **C**: However, principal component analysis gives no indication for clearly separated groups of units, neither for the different clusters gathered from hierarchical cluster analysis (**C1**) nor for the different PSTH types (**C2**). In both cases units establishing different groups tend to accumulate in different regions of the plot. Still, the different groups strongly overlap, especially in the centre of the plot. Thus, with respect to their physiological properties the AVCN neurons form a continuum rather than distinct groups.

The result of the hierarchical cluster analysis is shown as a dendrogram in figure 6A. Individual units are lined up at the bottom of the graph. With increasing linkage distance between units, successive branch points represent clusters of increasing size and dissimilarity. This dendrogram suggests five main clusters (I–V) of which one (cluster V) is characterized by a stronger separation from the rest.

For purpose of comparison, the values for all evaluated properties of the respective clusters are displayed in figure 6B and summarized in table 2. Cluster V is composed of 80 units which are particularly characterized by short inter-spike intervals (2.2 ms [1.8; 2.8]), a low variation of inter-spike intervals (0.9 ms [0.6; 1.4]), high maximum discharge rates (324 Hz [248; 428]), and low CF-thresholds (15 dB SPL [0; 25]). The units in this cluster never show waveforms with a presynaptic component.

**Table 2:** Physiological response properties of different clusters of AVCN units according to hierarchical cluster analysis using all evaluated properties.

response properties	Cluster^a^					Statistical analyses	
	I	II	III	IV	V	ANOVA	*Post-hoc* test^b^
**Frequency tuning curve**	**n = 54**	**n = 43**	**n = 20**	**n = 38**	**n = 78**		
CF [kHz]	21.8 (8.6; 31.9)	13.1 (1.7, 22.1)	14.7 (4.0; 27.8)	2.3 (1.2; 9.0)	5.8 (1.9; 16.3)	P<0.001	(I III) (II III IV) (II IV V)
TRS [dB]	45 (35; 60)	25 (20; 45)	35 (30; 48)	35 (25; 45)	15 (0; 25)	P<0.001	(I III IV) (II III IV) (V)
CF-BF [octaves]	0.09 (0; 0.56)	0 (0.02; 0.97)	2.17 (1.09; 2.72)	0 (0; 0.69)	0 (0; 0.28)	P<0.001	(I II IV V) (III)
BF-CF [Hz]	8 (0; 31)	8 (0; 21)	151 (92; 206)	6 (0; 26)	0 (0; 19)	P<0.00	(I II IV V) (III)
spontR [Hz]	1 (0; 13)	6 (0; 39)	0 (0; 9)	69 (32; 87)	9 (0; 30)	P<0.001	(I II III V) (IV)
maxR [Hz]	212 (171; 277)	138 (113, 171)	307 (238; 376)	186 (141; 268)	324 (248; 428)	P<0.001	(I III) (I IV) (III IV) (II)
Q_10_	2.47 (1.21; 3.81)	2.05 (1.38; 4.05)	2.36 (0.97; 5.43)	2.16 (1.79; 2.79)	1.72 (1.24; 2.97)	P = 0.219	(I II III IV V)
DR [dB]	36±14	28±13	48±18	27±16	42±13	P<0.001	(I) (II IV) (III V)
difference of slopes [octaves/10 dB]	0.13 (0.06; 0.27)	0.14 (0.05; 0,21)	0.49 (0.11; 0.93)	0.12 (0.05; 0.27)	0.10 (0.02; 0.24)	P<0.05	(I II II IV) (I II IV V)
**PSTH**	**n = 41**	**n = 43**	**n = 13**	**n = 14**	**n = 70**		
peak-over-total [%]	10.3 (8.5; 13.1)	3.9 (2.4; 5.8)	6.8 (3.4; 8.7)	12.8 (7.9; 17.6)	10.4 (8.4, 14.2)	P<0.001	(I IV V) (II III)
ISI mean [ms]	3.3 (2.6; 3,8)	4.5 (3.8; 5.4)	2.5 (1.9; 3.4)	2.9 (2.6; 3.2)	2.2 (1.9; 2.8)	P<0.001	(I III ) (II) (II IV V)
ISI S.D. [ms]	1.59 (1.12, 2.16)	2.74 (2.42; 3.49)	1.81 (1.11; 2.33)	1.61 (1.0; 2.07)	0.86 (0.58; 1.38)	P<0.001	(I II IV) (II) (IV V)
FSL mean [ms]	4.76 (3.70, 7.02)	4.06 (3.60; 4.91)	3.64 (3.16; 4.86)	3.81 (3.20; 4.27)	3.85 (3.30; 4.40)	P<0.001	(I II) (II III IV V)
FSL jitter [ms]	0.44 (0.25; 0.79)	0.80 (0.36; 1,43)	0.20 (0.12; 0.30)	1.39 (1.22; 1.86)	0.31 (0.15; 076)	P<0.001	(I II III IV) (II IV) (III V) (IV)
**SAM**	**n = 7**	**n = 19**	**n = 4**	**n = 3**	**n = 26**		
VS max	0.73±0.16	0.60±0.16	0.60±0.23	0.77±0.11	0.62±0.17	P = 0.251	(I II III IV V)
fmod VSmax [Hz]	200 (200; 200)	100 (50 200)	300 (110, 600)	200 (125; 275)	200 (50 200)	P = 0.671	(I II III IV V)
fmod VS>0,3 [Hz]	729±198	616±348	638±411	833±116	515±262	P = 0.255	(I II III IV V)
entr max	0.81±0.11	0.62±0.11	0.59±0.30	0.90±0.06	0.77±0.18	P<0.01	(I II III IV) (I III IV V)
fmod entr max [Hz]	200 (200; 200)	100 (50; 100)	200 (125; 250)	200 (200; 275)	200 (200; 300)	P<0.001	(I II III IV) (I III IV V)
**Waveform**	**n = 37**	**n = 36**	**n = 12**	**n = 14**	**n = 55**		
SNR	7.6 (6.7; 10.3)	6.3 (5.6; 7.6)	8.1 (6.4; 11.5)	9.6 (8.4; 12.0)	89 (6.6; 13.0)	P<0.001	(I II III V) (I III IV V)
t(AP) [ms]	0.31±0.06	0.29±0.07	0.27±0.07	0.27±0.04	0.27±0.07	P = 0.054	(I II III Iv V)

abbreviations explained in methods.

categorical values are described in text.

a Means ± S.D./Median (25%, 75%).

b cluster that do not significantly differ are within one parentheses.

Units in cluster I (n = 54) are characterized by long first spike latencies (4.8 ms [3.7; 7.0]), relatively high CFs (21.8 kHz [8.5; 31.9]), high thresholds (45 dB SPL [35]; [60]), and low spontaneous rates (0.8 Hz [0; 13.4]). Inhibitory sidebands were rarely seen (n = 2/54). The waveforms show two postsynaptic components in one third of the units (11/37), a P-component was never observed.

The typical parameter combination of units in cluster II (n = 43) is low peak-over-total values (0.04 [0.02; 0.06]), long inter-spike intervals (4.5 ms [3.8; 5.4]), and a large variation of the latter (2.7 ms [2.4; 3.5]), low maximum discharge rates (138 Hz [113; 171]), and high frequency selectivity (Q_10_ values: 2.05 [1.38; 4.05]). The signal-to-noise ratios of these units (6.3 [5.5; 7.5]) was the lowest in the entire sample. At the same time, most units in this cluster displayed complex waveforms; in one third of the units (11/34) prepotentials were recorded. In 9 other units (26%) the waveforms showed the two postsynaptic components.

Units in cluster III (n = 20) are characterized by low spontaneous (0.1 Hz [0; 8.8]), but high maximum discharge rates (307 Hz [238; 376]). The frequency tuning curves are mostly asymmetric, i.e. they showed large differences in the slope of the low and high frequency flanks (Δ: 0.5 octaves/10 dB [0.1; 0.9]), large differences between BF and CF (Δ: 2.2 octaves [1.1; 2.7]), and between the discharge rates at the respective frequencies (Δ: 151 Hz [92; 206]). The RLF are mostly monotonic (47%; 9/19) or monotonic- plateau (37%, 7/19) rather than non-monotonic (16%, 3/19). The dynamic ranges of these units (45 dB±18]) were the largest in the entire sample. Inhibitory sidebands were seen in only 2/20 cases (10%).

Cluster IV units (n = 38) show high peak-over-total values (0.13 [0.08; 0.18]), short first spike latencies (3.8 ms [3.2; 4.2]), but at the same time high jitter values (1.4 ms [1.2; 1.9]) and high spontaneous rates (68 Hz [36; 87]). These units had the highest incidence of inhibitory sidebands (89%, 34/38), and they responded well to fast fluctuations in signal amplitude.

In conclusion, the clusters establish well separated groups of units which differ significantly in a sizeable number of properties (see also table 2). Still, the different clusters show a strong overlap in the parameter fields. Thus, it may not be appropriate to think of the identified clusters as rigidly segregated classes of AVCN units.

To visualize the clusters in a lower number of dimensions a principal component analysis was performed. The principal component analysis linearly maps the high-dimensional representation to a lower dimensional space with its axes defined by weighted combinations of the original properties, the principal components. Clusters which are separated in the higher dimensions often remain separated in the lower dimensional projection.

The weights of the single units on the two first principal components (PCs) are plotted in figure 6C1. The properties that contribute mostly to PC1 are the maximum discharge rate (13%), the mean inter-spike interval (11%), and variation in inter-spike intervals (13%). The further left a unit lies on the PC1 scale, the higher was its maximum discharge rate (r = −0.75), the lower was its mean inter-spike interval (r = −0.67) and the variation of its inter-spike intervals (r = −0.76). But other properties correlate with the PC1 as well, e.g. the entrainment (r = −0.64) and the peak-over-total values (r = −0.55). The PC2 is influenced mostly by CF threshold (12%). The lower the value was on this PC scale, the higher was the unit's threshold (r = −0.60). All other properties contribute only little to PC2 (each less than 10%) and show only weak correlations (r<0.5).

In the two dimensional plot, the individual clusters occupy different regions, but the separation of the cluster is not perfect. Some units of cluster I are located in an area which contains primarily units of cluster III or V, some units of cluster IV in an area predominantly containing cluster II units, etc. Especially in the middle of the plot the clusters strongly overlap. Correspondingly, the first two PCs only constitute 28% (PC1: 17%, PC2: 12%) of the overall variance.

A potential problem of the analyses presented above is that missing values were replenished with the mean of the unit sample. This could be the reason for the accumulation of units in the centre. To address this issue, additional analyses were done with a reduced set of parameters, i.e. fewer properties.

#### Reducing parameters

The following ten properties were used in this analysis: peak-over-total, mean and standard deviation of the inter-spike intervals and the first spike latency, CF and CF threshold, spontaneous and maximum discharge rate, Q_10_ value and dynamic range. These physiological properties are easy to quantify from a limited number of extracellular recordings thus reducing the number of missing values. Consequently, all units with missing values were removed from analysis, which reduced the sample size from 233 to 174 units.

**Figure 7:**
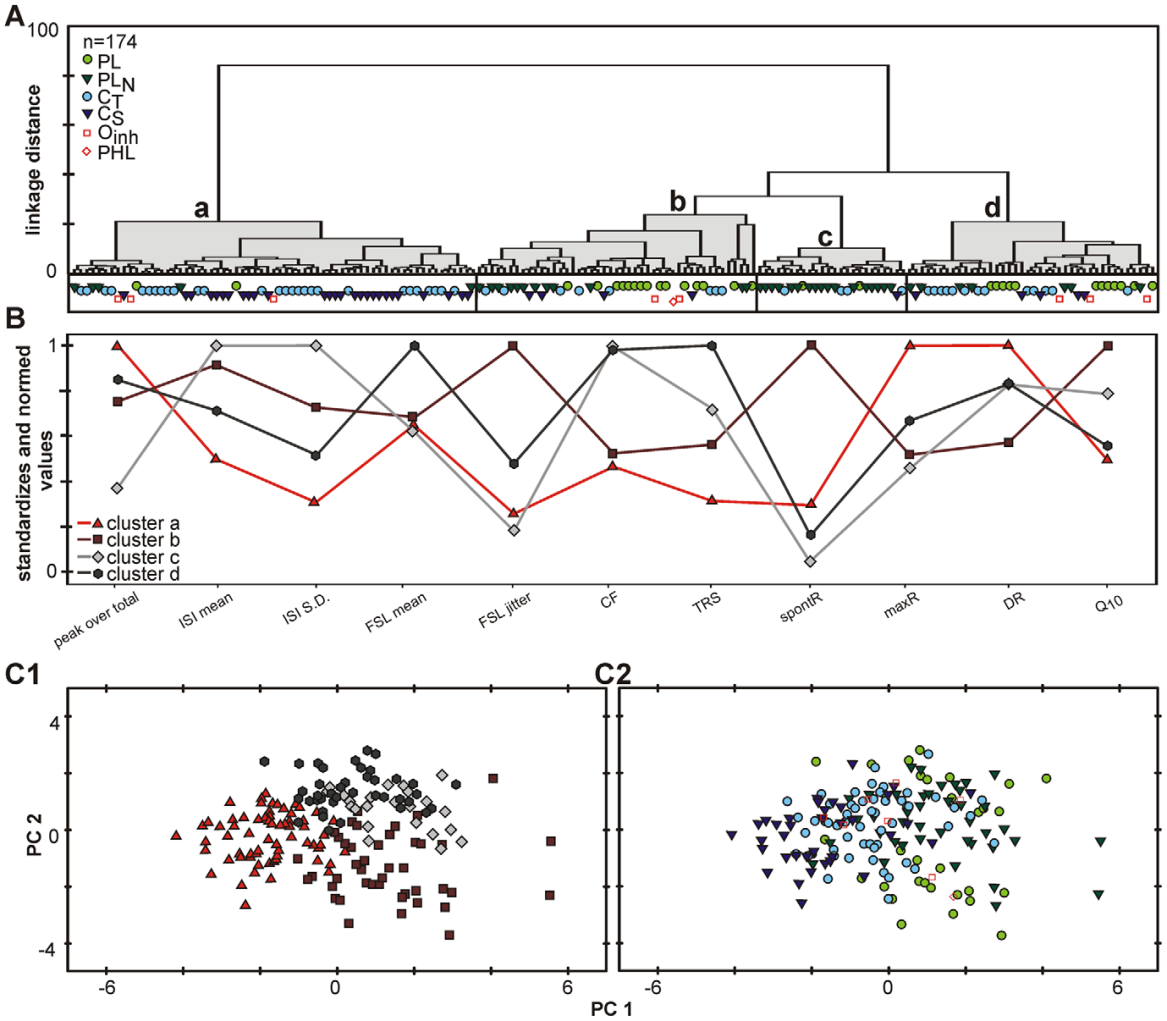
Cluster analyses based on a restricted set of parameters. See text for the reasons of the restriction. The parameter considered are indicated in B. Design of the graphs is the same as in figure 6. **A**: The present cluster analysis suggests a distinction of four clusters (a–d) with **B**: specific properties. Note that there is some correspondence between this restricted analysis and the analysis given in figure 6: Cluster ‘a’ relates to cluster V; ‘b’ to I, ‘c’ to II, and ‘d’ to IV. **C**: The principal component analysis arranges the units in one big coherent cluster. Units establishing different clusters (**C1**) and different PSTH types (**C2**) still could not be separated.

The results of this more restricted analysis are presented in figure 7. The dendrogram of the hierarchical-agglomerative procedure now results in four main clusters (a–d, figure 7A), but still the two analyses yielded partially comparable results (compare with figure 6A). In the restricted analysis, again one of the clusters shows a stronger separation from the rest. Here, it is cluster ‘a’ which shares some properties with cluster V in figure 5A. The units in this cluster show short inter-spike intervals and a low variation of it; high maximum discharge rates and low CF-thresholds. There are also partial overlaps of the other clusters with clusters of the extended analysis. Cluster ‘b’ has its counterpart in cluster I, and ‘c’ and ‘d’ in clusters II and IV, respectively.

**Figure 8:**
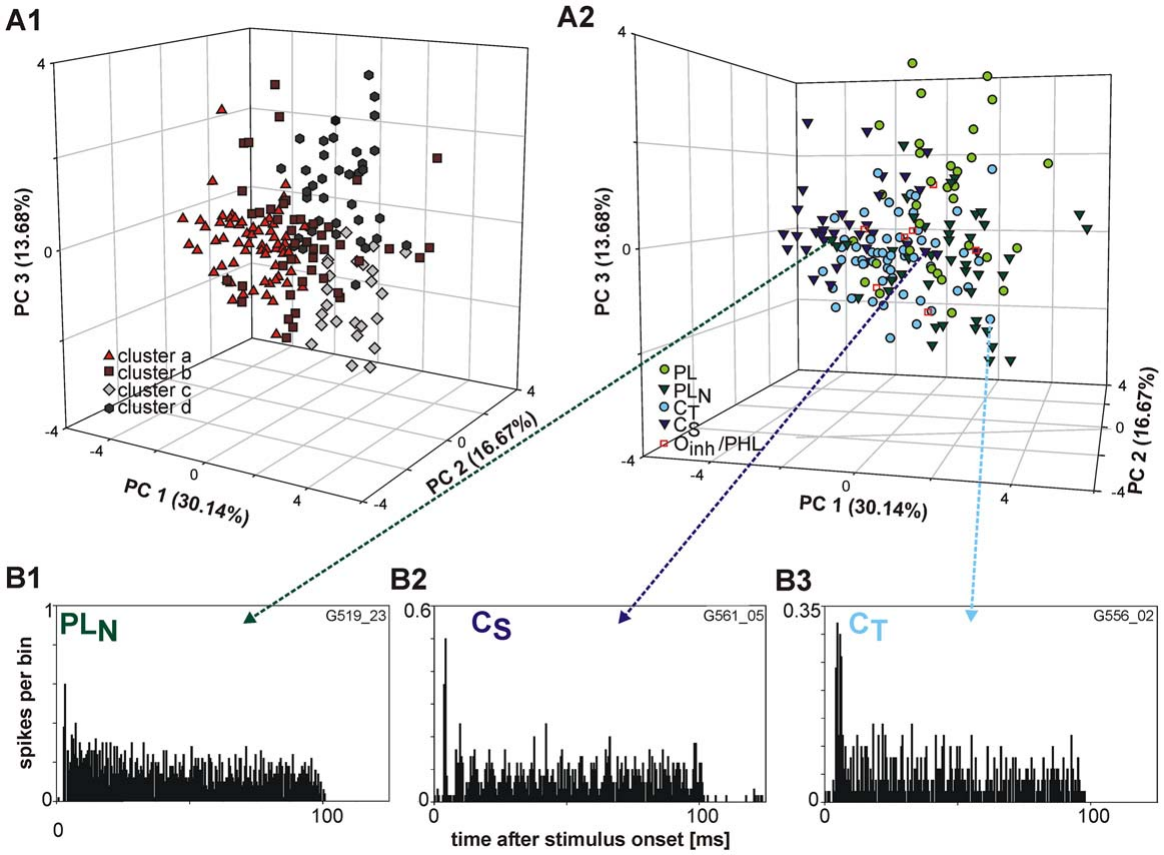
Principal component analysis employing three principal components and recheck of PSTH classification. Same sample of units (n = 174) and analysis as in figure 7. **A1**: Principal component analysis with assignment of the units to the different clusters gathered from hierarchical cluster analysis. **A2**: Principal component analysis with assignment of the units to the different PSTH types. Note that even a visualization based on the three dominant principal components does not indicate a clear separation of unit types. **B**: PSTHs of units in regions of the plot which are mainly occupied by other PSTH types, i.e. a PL_N_ (**B1**), a C_S_ (**B2**) and a unit which is not unambiguously to classify (**B3**). This unit was classified as C_T_, but it could also be a PL.

On average, the more restricted analysis shows smaller linkage distances within and larger between the clusters. Consequently, the resulting clusters are more homogeneous and differ more from each other. Still, the principal component analysis on the whole unit sample results in one big cluster of units instead of clearly separated groups (figure 7C1+2). We verified that this is not only an artefact of the projection into two dimensions by considering the results in three dimensions (figure 8A). Similar results for hierarchical clustering and principal component analysis were obtained when other parameter combinations were used, confirming the reliability of the depicted results.

#### Reconsidering the classification based on PSTH-types

To evaluate potential links between the hierarchical cluster analysis and the classification based on the PSTH types, we assessed how the PSTH types fit into the clusters obtained by the previous analysis. For this, the distribution of the different PSTH types in the multidimensional parameter space was analysed. None of the clusters contains exclusively one PSTH type, but the distribution of the PSTH types differs between the clusters (figures 6A+7A). Cluster I mainly includes PL (30%, n = 16), PL_N_ (30%, n = 16), and C_T_ (19%, n = 10) units, while the proportion of C_S_ (11%, n = 6), O_inh_ (6%, n = 3), and phase-locking units (3%, n = 2) was low. In cluster II the majority of units have a PL_N_ (52%, n = 22) or a PL PSTH (24%, n = 10). In cluster III 58% (n = 11) of the units respond with a C_T_ pattern and 16% (n = 3) with PL and PL_N_ respectively. Cluster IV mainly consists of PL (45%, n = 17), O_inh_ (24%, n = 9) and C_T_ (18%, n = 7) units. The dominant response patterns in cluster V are C_T_ (38%, n = 30) and C_S_ (36%, n = 29).

In the principal component analysis, the PSTH types show a stronger overlap than the clusters obtained from hierarchical clustering (figures 6C2+7C2+8A2). Still, units of different PSTH types tend to occupy different regions of the parameter space. The *chopper* units generally were found at the upper left side of the plot, and PL and PL_N_ units more at the right side. Moreover, the PL units tend to be located in the upper quadrant and the PL_N_ in the lower one.

To exclude the effect of possible misleading PSTH classifications caused by outliers, we checked the temporal response patterns of those units that were located separated relative to units of the same PSTH type (figure 8B). We could confirm most of our previous classifications, but few units we found with PSTHs that are not unambiguously to classify. For example the unit in figure 8B3: This unit could either be classified as PL unit, bases on the strong onset and the following sustained rate which is comparable to auditory nerve fiber responses, or as CT unit since there are two sharp peaks in the onset component and the CV value is above 0.35. This kind of PSTH shapes could somehow described as mixture of different ‘classic’ PSTH types.

In conclusion, it can be stated that based on their physiological response properties, AVCN units can better be described as occupying specific areas in a coherent multidimensional parameter space, rather than forming clearly separable groups of units.

## Discussion

The present report provides the first comprehensive evaluation of the physiological properties of AVCN neurons in the Mongolian gerbil in vivo. More important, this study re-evaluates the question of classifying AVCN neurons. Based on our present results, we conclude that units in the AVCN form a continuum concerning their physiological response properties, with each unit having a fingerprint-like combination of several properties. Thus, the present results challenge the conventionally held idea of separable classes of AVCN units. However, we show that units, which stand out by their prototypic physiological response properties, are located towards the extremes of this continuum. The significance of a classification lies in the fact that the AVCN is the starting point of distinct ascending auditory pathways. Hypotheses about specific auditory processing along these pathways must consider the functional organisation of the AVCN. We will discuss possible causes for such a continuum and the consequences for the functional differentiation of the auditory pathways.

### Limitations of the study

In the first part of the present study we demonstrate that our sample of AVCN units is in most respects comparable to those previously described in other mammalian species [7]–[9], [28]. There are only minor deviations, for example the relatively long latencies in PL units. Based on the (somewhat limited) data we have from these units for lower-level stimulation, we can rule out that level dependence or nonlinear RLFs are the determining factors for these differences. Also, it is unlikely that differences in spontaneous rates are casual for this result, since PL units tended to have higher spontaneous rates than the other cell classes (Figure 4C1), and such higher rates would cause a trend for latency shifts towards lower values. Finally, species differences can be ruled out, since Frisina et al. [21] also observed shortest latencies for the PL units in the gerbil. However, in other respects the PL units are comparable with those previously described, but we could not finally rule out distortion effects on our analysis.

Generally, it cannot be excluded that certain cell class distinctions would only become apparent, if a different or an even wider set of parameters/conditions were included in the cluster analysis. The limited recording time in *in vivo* single unit electrophysiology makes it necessary to seek a compromise between the use of a standardized stimulus repertoire and extensive variations of stimulus parameters. Still, in future studies it could be more revealing to analyze responses to more complex stimuli (i.e. broadband stimuli) or to study the system under continuous activation, e.g. via the use of long, natural stimuli. Such an approach would require the use of more general neuronal characterizations, e.g. spectro-temporal receptive fields or multilinear models as recently employed in the medial nucleus of the trapezoid body [37]. Also, PSTHs were only collected at one common sound level (80 dB SPL) for the reasons explained in the methods section. If PSTH types were to systematically change for different relations of sound level and rate-level function of a given neuron, some cells which currently fall far away from the majority of units in their corresponding PSTH type might have been classified more consistently.

However, for a subset of our neurons (n = 60) we also collected the PSTH at 20 dB above their threshold. The PSTH shape for low and high intensity stimulation was very similar when normalized for mean rate (r = 0.85). Similarly, the inter-spike interval histograms for the low and high intensity stimulation were highly correlated (r = 0.92). Therefore, including this low level stimulation into the cluster analyses was unlikely to provide a better distinction between the cells.

Evidently not all possible parameters were included in the present analysis (e.g. phase-locking filter type could have been included if more data were available), but still we tried to include a wide range that could aid the classification. Additionally, we reran the cluster analysis and the PCA with different combinations of the acquired parameters to exclude distortion effects of any single parameter. None of the respective combinations revealed distinct physiological neuron types in our data sample.

To support the physiological data, it would also be useful to have additional data about cell morphology and projection pattern. Unfortunately, single-unit labeling is not easy to implement *in vivo* for a substantial number of units. At the same time, including it would defy the purpose of achieving a physiological classification.

The present focus on the Mongolian gerbil as a model system for human (low frequency) hearing inherits a potential caveat, as recent studies have reemphasized the potential deterioration of auditory processing due to microcysts forming also in the cochlear nucleus of the gerbil [38]–[40]. The functional consequences of this gerbil-specific condition, which develops mostly after sexual maturity, are not clear. However, the present study used comparatively young gerbils (2–4 months) to avoid potential effects of altered CN morphology. Given that the Mongolian gerbil is a well-introduced animal model, future studies might consider the use of even younger specimen or switching to other species.

### Variability of response properties of AVCN neurons

When dealing with physiological classifications of AVCN units *in vivo*, earlier studies mostly relied on the units' temporal response properties to pure tone bursts as visualized in the PSTHs. The present sample of AVCN units recorded in the Mongolian gerbil fits well into the ‘classical’ PSTH types described for a number of mammalian species (gerbil: [17], [21], [22]; cat: [7]–[9], [33], guinea pig: [41], [42]). However, the analysis presented here is based on a larger number of physiological features, all acquired from the same sample of AVCN units. Hence, the assessment of potentially distinct physiological neuron types rested on a broader base.

With respect to their morphology, AVCN neurons are subdivided in at least three different classes; spherical and globular bushy cells and stellate cells (review: [3], [6]). Several studies attempted to relate these different groups of neurons to physiological response types yielded by acoustic stimulation (gerbil: [17]; rat: [13], [18]; guinea pigs: [43]; cat: [10]–[12], [14], [15], [19], [44]). Most studies agree that PL responses are best attributed to spherical bushy cells, PL_N_ responses to globular bushy cells, and any kind of *chopper* responses to stellate cells. However, the same studies reported a number of cases where such a simple agreement between PSTH type and morphological cell type does not hold. Especially globular bushy cells are reported to show a variety of different response patterns. Besides the PL_N_ pattern, PL and various onset patterns are found in this cell type [12], [15], [19], [44]. Similarly, also for spherical bushy cells and stellate cells a variety of PSTH types have been described [43], [44]. Thus, either the correspondence between morphology and PSTH types of AVCN neurons is very complex, or the PSTH alone is not sufficient to establish the quested structure-function relationship, or the two modes of classification are mostly independent from each other.

A correlation of PSTH types with a number of other physiological properties, such as spontaneous rate and first spike latency, has been suggested in previous studies [7]–[9], [28]. But considering these correlations, it was not possible to separate the units into non-overlapping groups. This problem has already been addressed in a number of earlier studies [9], [28], [42] which attempted to develop different schemes for classifying AVCN units based on PSTH types considering different features of the unit's response properties (e.g. spiking regularity and response latency). Still, the authors of these studies admitted that the “continuous filling of the parameter map” [42] and the “overlap of the characteristics of neighbouring unit types” suggest that the classification schemes ”do not provide absolute borders between response types” [28].

In contrast to previous studies, our study is based on a larger repertoire of physiological parameters. Additionally, we avoided to make any assumptions on the significance of specific parameters. By performing cluster analyses, an unbiased analysis was possible. Even with this precautionary measure it was not possible to identify clearly separated physiological unit types. The units rather have a fingerprint-like combination of several properties and show a wide distribution in the multidimensional parameter space. While this does not preclude a correlation of some physiological properties across units, it appeared hard to establish an unambiguous structure-function relationship on this basis.

### Possible causes for a physiological continuum

It is widely accepted that the morphologically defined AVCN neuron types [4]–[6] establish the origin for several distinct ascending auditory pathways. This is tantamount to distinct projection patterns of the neuron types [3]. Also, in *in vitro* preparations the units can be differentiated with respect to their basic physiological properties [45]–[47].

Hence, the question arises, what could cause the higher degree of variability in physiological characteristics across different AVCN units *in vivo*? The more uniform features obtained *in vitro* might relate to the reduced complexity of the system. *In vivo* there are several levels at which the units' responses can be modulated to show a wider range of response characteristics. With respect to the afferent input, some variability already exists in the activity of auditory nerve fibres [48]–[52]. Next, the degree of convergence of auditory nerve fibres onto single CN neurons varies, ranging from 3–5 in spherical bushy cells [53]–[56] to 9–69 in globular bushy cells [57]–[59]. Even higher degrees in convergence were reported for stellate cells [53], [60]–[63]. Furthermore, variations of the synaptic strength of auditory nerve fibers on AVCN neurons can determine the response properties of the postsynaptic cells [62]–[66]. Additionally, there are secondary excitatory and inhibitory inputs [57], [67] which also can affect the response. Furthermore, postsynaptic properties can determine a unit's response as well. The units show a certain degree of variability in their morphology, first of all the size of their somata and dendritic branching patterns [3], [68]. Moreover, the neurons differ in the expression of specific subsets of receptors, ion channels and membrane proteins [68]–[75] which influence their membrane characteristics. It is conceivable that a multifold of these pre- and postsynaptic influences contribute to the establishment of a continuum in response properties.

### Consequences for concepts of auditory pathways and functional implications

As discussed above, it cannot be ruled out that future studies with a different set of stimuli will provide a basis for an unambiguous classification. However, if the continuum of physiological properties cannot be resolved, the notion of classifying AVCN neurons on a physiological level would have to be reconsidered. Classification schemes have a great value in organizing our understanding of the system, but they do not have to reflect actual boundaries. While such classifications are often based on the description of prototypical representatives of each class, many cells appear to fill the parameter space between the prototypes and only fit ‘more or less’ into the classification. In the ‘classical’ concept, the AVCN is composed of a set of distinct cell classes, each of which occupies a well-defined location in the space of all possible combinations of properties. While such a representation is favourable from the perspective of a human observer (since it allows an easier conceptualization), it need not to be favourable from a more general coding perspective. As has been shown theoretically (e.g. liquid state machines) [76], that a broad representation of various parameter combinations considerably simplifies the task of extracting many complex aspects of the original signal. In this sense the presently observed continuum of cell properties could be advantageous for processing in subsequent higher-order nuclei of the ascending auditory system.
